# Arthritis augments breast cancer metastasis: role of mast cells and SCF/c-Kit signaling

**DOI:** 10.1186/bcr3412

**Published:** 2013-04-11

**Authors:** Lopamudra Das Roy, Jennifer M Curry, Mahnaz Sahraei, Dahlia M Besmer, Amritha Kidiyoor, Helen E Gruber, Pinku Mukherjee

**Affiliations:** 1 Department of Biology, University of North Carolina at Charlotte, 9201 University City Blvd., Charlotte, NC 28223, USA; 2 Department of Pharmacology, Yale University, 10 Amistad St., New Haven, CT 06519, USA; 3 Department of Orthopedic Surgery, Carolinas Medical Center, 1543 Garden Terrace, Charlotte, NC 28232, USA

## Abstract

**Introduction:**

Breast cancer remains the second leading cause of cancer-related deaths for women in the United States. Metastasis is regulated not only by intrinsic genetic changes in malignant cells, but also by the microenvironment, especially those associated with chronic inflammation. We recently reported that mice with autoimmune arthritis have significantly increased incidence of bone and lung metastasis and decreased survival associated with breast cancer. In this study, we evaluated the mechanism underlying the increased metastasis.

**Methods:**

We used two mouse models; one that develops spontaneous autoimmune arthritis (SKG mice) injected with metastatic breast cancer cells (4T1), and another that develops spontaneous breast cancer (MMTV-PyV MT mice) injected with type II collagen to induce autoimmune arthritis. Mast cell levels and metastasis were monitored.

**Results:**

First, we confirmed that breast tumor-bearing arthritic mice have a significantly higher incidence of bone and lung metastasis than do their nonarthritic counterparts. Next, we showed increased recruitment of mast cells within the primary tumor of arthritic mice, which facilitates metastasis. Next, we report that arthritic mice without any tumors have higher numbers of mast cells in the bones and lungs, which may be the underlying cause for the enhanced lung and bone metastases observed in the arthritic mice. Next, we showed that once the tumor cells populate the metastatic niches (bones and lungs), they further increase the mast cell population within the niche and assist in enhancing metastasis. This may primarily be due to the interaction of c-Kit receptor present on mast cells and stem cell factor (SCF, the ligand for ckit) expressed on tumor cells. Finally, we showed that targeting the SCF/cKit interaction with an anti-ckit antibody reduces the differentiation of mast cells and consequently reduces metastasis.

**Conclusion:**

This is the first report to show that mast cells may play a critical role in remodeling not only the tumor microenvironment but also the metastatic niche to facilitate efficient metastasis through SCF/cKit interaction in breast cancer with arthritis.

## Introduction

In 2012, an estimated 229,060 new cases of breast cancer (BC) are expected to be diagnosed in women, and about 39,920 women are expected to die of the disease because of metastasis [1]. The most common site of metastasis is the bone, and bone-disseminated BC is incurable. Studies in the past 10 years have begun to elucidate the role of cytokines/chemokines and bone remodeling during BC-associated bone metastasis [2-5]. Several studies demonstrated that sites of chronic inflammation are associated with the establishment and growth of tumor cells [2]. One such common inflammatory condition in humans is autoimmune arthritis (AA), which causes inflammation and deformity of the joints, as well as increased cellular infiltration and inflammation of the lungs [6]. Although AA and BC are different diseases, some of the underlying molecular processes that characterize AA also affect cancer progression and metastasis. The bones and lungs not only are the most common sites of chronic inflammation linked to AA, but also are frequent sites of BC metastasis. In addition, epidemiologic studies indicate that BC patients with rheumatoid arthritis (RA) have poor prognoses and higher mortality in comparison with BC patients without RA [7]. Thus, an understanding of the molecular mechanisms and factors that facilitate BC-associated metastasis in arthritic conditions is highly significant.

We reported that mice with AA have a significantly increased incidence of bone and lung metastasis and decreased survival associated with BC [8,9]. We further demonstrated that critical proinflammatory factors (interleukin-17 (IL-17), IL-6, matrix metallopeptidase-9 (MMP-9), cyclooxygenase-2 (COX-2), vascular endothelial growth factor (VEGF), and tumor necrosis factor-alpha (TNF-α)) triggered by AA serve as chemoattractants for recruitment, retention, and proliferation of BC cells in the bones and lungs [8,9]. Interestingly, these mentioned proinflammatory factors are produced by mast cells (MCs), which can be activated by tumor-derived SCF [10]. SCF is a cytokine/ligand that binds to the c-Kit receptor (CD117) on MCs. MCs are highlighted as a major regulator of inflammation [11,12]. It is also reported that tumor-infiltrating MCs remodel the tumor microenvironment and promote tumor growth [10].

Thus, we hypothesized that the enhanced BC-associated metastasis in the arthritic mice may be due to the activation of MCs via the SCF/c-Kit interaction and that disrupting this interaction may reduce metastasis. We have used two relevant transgenic models that mimic the human disease: one that develops spontaneous AA (SKG mice) bearing orthotopic 4T1 tumors in the mammary fat pad and another that develops spontaneous mammary gland tumors (PyV MT mice) induced to develop AA.

The SKG mice carry a mutation of the gene encoding an SH2 domain of ZAP-70, a key signal-transduction molecule in T cells, and spontaneously develop T cell-mediated chronic AA [13]. The mutation impairs positive and negative selection of T cells in the thymus, leading to thymic production of arthritogenic autoimmune CD4^+ ^T cells. The mice succumb to symmetrical joint swelling, beginning in the small joints of the digits and progressing to the larger joints. This is accompanied by severe synovitis with formation of pannus that invades and erodes the adjacent cartilage and subchondral bone. Genetic deficiency of IL-6, IL-1, or TNF-α inhibits development of AA in SKG mice [14], similar to the effects of anticytokine therapy in human arthritis [15]. These mice, when injected with the 4T1 tumor cells in the mammary fat pad, develop metastatic BC. The clinical and immunopathologic characteristics of AA in these mice make the strain a suitable model for testing our hypothesis.

The PyV MT mice are an established model of metastatic mammary gland tumors in a C57BL/6 background [16,17]. These mice carry the polyoma middle-T antigen driven by the MMTV promoter. The mice spontaneously develop mammary gland hyperplasia at puberty and progress to develop carcinoma *in situ *and invasive adenocarcinoma by 24 weeks of age. When injected with type II collagen (CII), the PyV MT mice develop typical signs of RA. The ensuing pathogenesis includes synovial hyperplasia, mononuclear cell infiltration, and cartilage degradation [18,19]. This model is clinically relevant, as tumors are phenotypically similar to the metastatic human breast tumors [16,17].

We first report the levels of MCs in the primary tumors and metastatic niches (bone and lung). Next, we demonstrate the significance of the SCF/cKit-receptor signaling in inducing metastasis. Finally, we report that interrupting the SCF/cKit receptor substantially reduces BC-associated metastasis to the bone and lung.

## Materials and methods

### Mice

SKG mice were established from a closed breeding colony of Balb/C mice [20]. At about 3 months old, mice were injected with 1 × 10^5 ^4T1 cells (in 100 μl of PBS) in the mammary fat pad. Balb/C mice were used as the nonarthritic controls. After 35 days of tumor challenge, all mice were killed, and analysis was conducted.

PyV MT oncogenic mice were originally a gift from Dr. W. J. Muller (McGill University, Toronto, Ontario, Canada) [17]. The PyV MT mice were bred to be congenic on the C57Bl/6 background and were used in several of our prior publications [9,21-24]. Genotyping of these mice was conducted as described previously [21].

All mice were bred and maintained in specific pathogen-free conditions in the UNCC Animal Facility. All experimental procedures were conducted according to Institutional Animal Care and Use Committee guidelines. All protocols were approved by the UNCC Internal Animal Care Review Committee (IACUC ID: 08-036.0 and 11-015.0).

### Cell lines

The 4T1 cells were purchased from The American Type Cell Culture Collection (Manassas, VA, USA). This highly metastatic BC cell line is derived from a spontaneously arising Balb/C mammary tumor. Cells were maintained in complete RPMI [8]. 4T1 tumors resemble human late-stage metastatic BC [25-27].

The PyV MT cell lines were generated from PyV MT tumors and cultured as previously described in complete DMEM [9].

### Induction of arthritis

The PyV MT mice were injected with 50 μl of 2-mg/ml CII (MD Biosciences, St. Paul, MN, USA) in CFA (Difco Laboratories, Detroit, MI, USA) intradermally about 1.5 cm distal from the base of the tail at 12 weeks of age. Fifty-sixty percent of mice developed arthritis within 15 to 30 days after collagen injection [28]. All mice were killed at 22 weeks, and analysis was conducted.

### Flow cytometry

BMMCs were stained with FITC rat anti-mouse CD117 antibody (BD Pharmingen, San Diego, CA, USA). Acquisition was performed on a FACS Fortessa Cytometer, and analysis was performed by using the Flow Jo program.

### Western blot and antibodies

We performed BCA assay to load equal quantities of tumor lysates onto sodium dodecylsulfate-polymerase chain reaction (SDS-PAGE) gels. SCF and β-actin antibodies were used at 1:200 dilution (Santa Cruz Biotechnology, Santa Cruz, CA, USA) and were used according to manufacturer's recommendations.

The densitometric analyses of immunoblots as shown in Additional file 1 were performed by using NIH Imaging program. Results are presented as mean values of arbitrary densitometric units corrected for background intensity and normalized to the expression of β-actin.

### Histology

Lungs, bones, and tumor sections were processed as previously described [8,9]. Paraffin-embedded blocks were prepared, and 4-μm-thick sections were cut for hematoxylin/eosin (H&E) and pancytokeratin staining. The pancytokeratin antibody was purchased from Santa Cruz and used at 1:50 dilution by following the same protocol as described in our previous publications [8,9]. To establish the expression of MCs on the tumors, lungs, and bones, anti-mast cell tryptase antibody (ab134932) was purchased from Abcam, Cambridge, MA, USA.

To establish further the expression of MCs, toluidine blue purchased from Fluka Analytical (Exeter, Devon, UK) distributed by Sigma-Aldrich (Sigma Aldrich, St. Louis, MO, USA) was used as directed by the manufacturer. All images were taken by using Olympus DP71 light microscopy.

### Immunofluorescence

Cells were plated on chamber slides and grown to 75% confluency followed by immunofluorescence (IF) staining with SCF rabbit antibody at 1:200 dilution and Alexafluor 594 donkey anti-rabbit (Invitrogen) secondary at 1:1,000. Staining was performed as previously described [29]. Slides were examined under Olympus FV1000 fluorescence microscopy, and pictures were taken at 400× magnification.

### Generation of bone marrow-derived mast cells

BM cells were harvested from femurs of mice, and 2 × 10^6 ^cells were cultured, as previously described [10]. The cells were cultured in the presence of 10 ng/ml IL-3 and 50 ng of recombinant murine SCF (PeproTech, Rocky Hill, NJ, USA), and the nonadherent cells were passaged every 3 days. Four weeks later, the cells were used as MCs for experiments [10,30]. The MCs were identified and counted in three different ways: (a) light microscopy: we counted the MCs with a hemocytometer and used trypan blue to detect any dead cells [30]; (b) flow cytometry: BMMCs were stained with FITC rat anti-mouse CD117 antibody, and the percentage of MCs was analyzed with flow cytometry. CD117 is the cKit receptor present on mast cells; and (c) toluidine staining [30]. We counted five fields of toluidine-positive BMMCs generated from BM derived from each mouse.

### Isolation of viable circulating BC cells

Blood samples (0.5 to 1.0 ml) were obtained by cardiac puncture from individual tumor-bearing animals by using heparin as the anticoagulant and processed as described previously [29,31].

Cells were cultured in complete DMEM for 21 days.

### Migration assays

SCF expressing 4T1 and PyV MT cells were plated over transwell inserts in the upper chamber (BD Biosciences, San Diego, CA, USA) and permitted to migrate toward MCs in the lower chamber for 15 hours. Percentage migration was determined as described earlier [29]. We added neutralizing SCF antibody (R&D Systems, Minneapolis, MN, USA) to tumor cells or anti-cKit antibody (eBioscience, San Diego, CA, USA) to the MCs as independent groups for 12 hours and analyzed the migration of BC cells toward MCs.

### X-ray imaging

The Pix array 100 x-ray machine (Bi Optic Inc, Santa Clara, CA, USA) was used for bone imaging, as described [32].

### Treatment schema

When the tumors were about 5 mm in length (after about 6 days of 4T1 injections), mice were injected (IP) with neutralizing rat anti-mouse cKit-receptor antibody (50 μg) (eBioscience) or 20 μg goat anti-mouse-SCF antibody (R&D Systems, Minneapolis, MN, USA) once a week for 4 weeks. The mice were euthanized 24 hours after the last injections at about 35 days after tumor challenge. For treatment experiments, 4T1 cells transfected with green fluorescent protein (4T1-GFP) were injected.

### Statistical analysis

Data were analyzed by using GraphPad software. Results are expressed as mean ± SEM and are representative of three or more separate experiments. Comparison of groups was done by using one-way or two-way ANOVA followed by the Bonferroni posttest for multiple comparisons (*P < 0.05, **P < 0.01, ***P < 0.001). The Student *t *test was used for comparing the level of significance between two experimental groups.

## Results

### Significant increase in primary tumor burden as well as lung and bone metastasis in the arthritic versus nonarthritic mice with breast cancer

**Figure 1:**
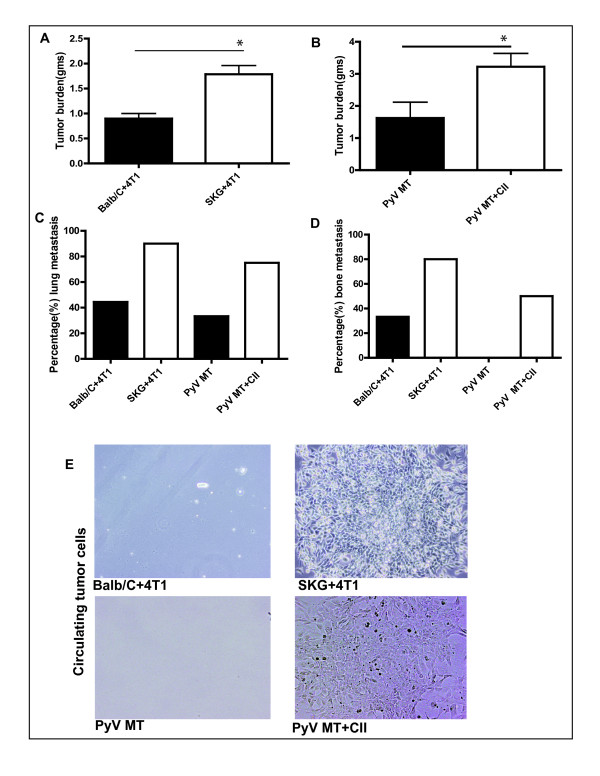
**Higher tumor burden coupled with higher metastasis in arthritic versus nonarthritic breast cancer-bearing mice**. **(A, B) **Significant increase in tumor burden in SKG versus Balb/C (*P < 0.05) and arthritic PyV MT versus PyV MT (*P < 0.05) mice. **(C, D) **Percentage of lung and bone metastasis, respectively. **(E) **Light-microscopic images of circulating tumor cells cultured from peripheral blood of arthritic versus nonarthritic tumor-bearing mice. (A through E, 10 mice).

We first demonstrated that the 4T1 tumor burden is significantly higher in the arthritic SKG versus the nonarthritic Balb/C mice (Figure 1A). Second, we substantiated this finding in the PyV MT mice with higher tumor burden in the arthritic PyV MT versus the nonarthritic PyV MT mice (Figure 1B). Third, metastases to the lungs and bones were compared. We report that nine of 10 SKG mice showed lung metastasis, whereas only four of 10 Balb/C mice showed metastasis (Figure 1C). Similarly, seven of 10 arthritic PyV MT mice showed lung metastasis, whereas only three of 10 nonarthritic Py VMT mice showed the same (Figure 1C). Similar trends were observed with bone metastasis, with eight of 10 SKG mice developing bone metastasis, whereas only three of 10 Balb/C developing the same (Figure 1D). Likewise, five of 10 arthritic PyV MT mice developed bone metastasis, whereas none of the nonarthritic PyV MT mice developed bone metastasis (Figure 1D).

Circulating tumor cells (CTCs) are being aggressively explored as a prognostic tool and a measure of metastasis and response to therapy [29,31]. One way to evaluate CTCs in mice is to culture the blood and detect colonies of tumor cells. We detected higher levels of CTCs (that formed colonies within 3 weeks of culture) in the blood of the tumor-bearing arthritic mice compared with the blood from the tumor-bearing nonarthritic mice (Figure 1E). The data corroborate our previous studies [8,9] and further establish that the proinflammatory microenvironment created in bones and lungs by AA is highly conducive for BC cells to form metastases.

### Mast cell population in the tumor microenvironment and metastatic niche

**Figure 2:**
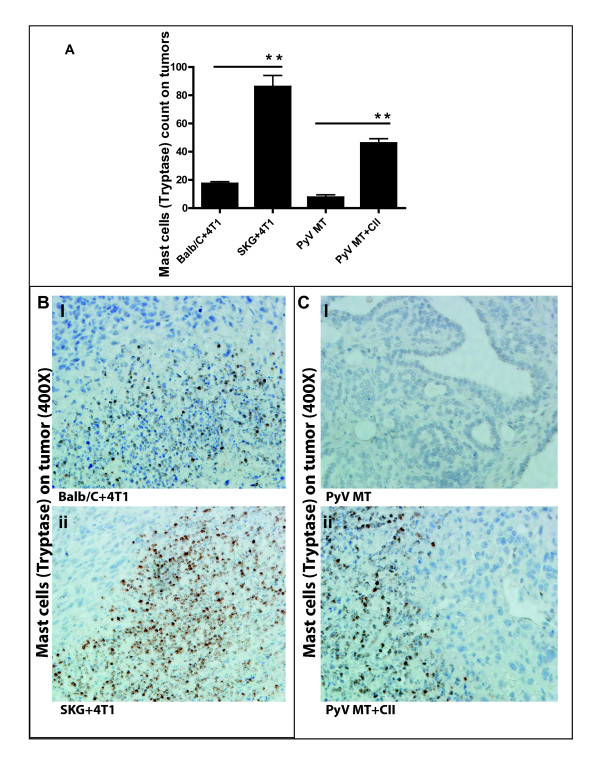
**Expression of mast cells (MCs) tryptase staining on tumors**. **(A) **Significant increase in MCs tryptase count in the tumors of arthritic SKG mice challenged with 4T1 cells versus nonarthritic Balb/C mice injected with 4T1 cells (**P < 0.01) and in arthritic PyV MT mice induced with CII versus nonarthritic PyV MT mice (**P < 0.01). Representative images of tryptase staining in **(B, C): **tumors taken at 400× magnification (six mice per experimental group and 10 fields per section). Brown staining represents MCs tryptase expression.

**Figure 3:**
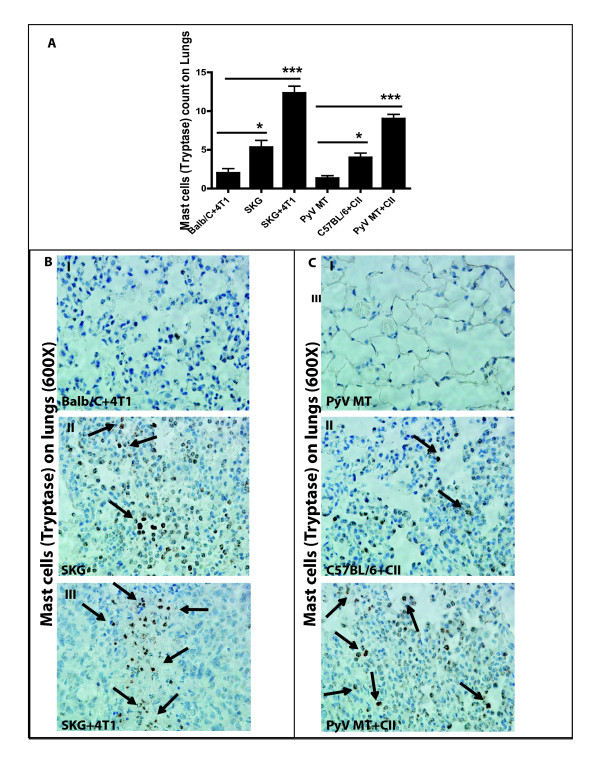
**Expression of mast cells (MCs) tryptase staining on lungs**. **(A) **Significant increase in MCs tryptase count in the lungs of arthritic SKG mice challenged with 4T1 cells versus nonarthritic Balb/C mice injected with 4T1 cells (***P < 0.001) and in arthritic PyV MT mice induced with CII versus nonarthritic PyV MT mice (***P < 0.001). Significant increase in MCs tryptase also is observed in the lungs of arthritic SKG mice with no tumor versus Balb/C mice with breast cancer (BC; *P < 0.05). Similarly, we see the same trend in arthritic C57BL/6 mice induced with CII versus spontaneous BC model of PyV MT mice with no arthritis (*P < 0.05). Representative images of tryptase staining in **(B, C) **lungs taken at 600× magnification (six mice per experimental group and 10 fields per section). Brown staining represents MCs tryptase expression.

**Figure 4:**
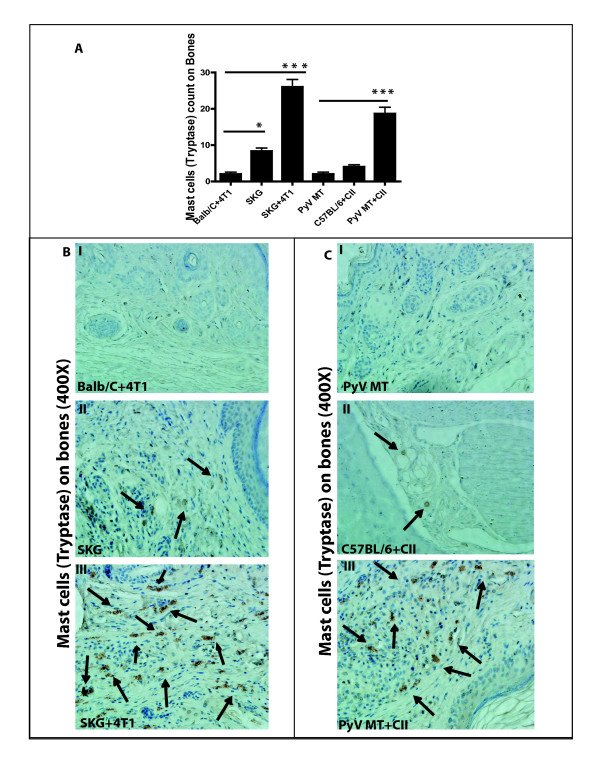
**Expression of mast cells (MCs) tryptase staining on bones**. **(A) **Significant increase in MCs tryptase count in the bones of arthritic SKG mice challenged with 4T1 cells versus nonarthritic Balb/C mice injected with 4T1 cells (***P < 0.001) and in arthritic PyV MT mice induced with CII versus nonarthritic PyV MT mice (***P < 0.001). Significant increase in MCs tryptase also is observed in the bones of arthritic SKG mice with no tumor versus Balb/C mice with BC (*P < 0.05). Similarly, although not significant, we see the same trend in arthritic C57BL/6 mice induced with CII versus a spontaneous BC model of PyV MT mice with no arthritis. Representative images of tryptase staining in **(B, C) **bones taken at 400× magnification (six mice per experimental group and 10 fields per section). Brown staining represents MCs tryptase expression.

We first assessed the expression of MCs in the tumor and metastatic niches (bone and lung). Tryptase is the most abundant secretory granule-derived serine contained in MCs that has been used as a marker for MC activation. In addition, tryptase has been shown to be a sensitive and specific marker for the localization of MCs in tissues [33-35]. By doing MC tryptase staining by IHC, we detected significantly higher numbers of MCs in the tumors of arthritic versus nonarthritic mice with BC (Figure 2A). We detected infiltration of MCs in the tumors of SKG versus Balb/C mice with BC (Figure 2B) and in the PyV MT mice induced with arthritis versus PyV MT mice with no arthritis (Figure 2C). We further identified significantly higher levels of MCs in the sites of metastasis: lungs (Figure 3) and bones (Figure 4) of arthritic versus nonarthritic mice in both (SKG and PyV MT) models. Thus, data suggest that an increase in MC migration and activation occurs within the tumor as well as in the metastatic niches, as indicated by the increased MC expression.

**Figure 5:**
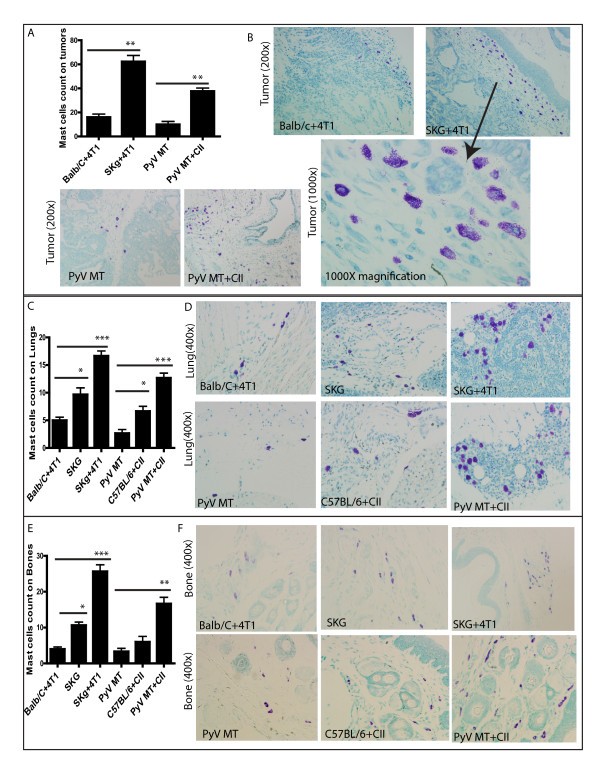
**Toluidine staining to confirm mast cell (MC) expression**. Significant increase in MCs toluidine count in arthritic mice with BC (SKG+4T1 and PyV MT+CII) versus nonarthritic (Balb/C+4T1 and PyV MT) BC models in **(A) **tumors (**P < 0.01), **(C) l**ungs (***P < 0.001), and **(E) **bones (***P < 0.001; **P < 0.01). Significant increase in MCs toluidine staining was also observed in arthritic control (SKG and C57BL/6+CII) mice with no tumor as compared with nonarthritic (Balb/C mice with BC and spontaneous BC PyV MT mice) in (C) lungs (*P < 0.05) and (E) bones (*P < 0.05). We observed the same trend of MC expression with toluidine staining in tumors, lungs, and bones of arthritic versus nonarthritic BC models as in MCs tryptase staining (Figures 2 through 4). Representative images of toluidine staining in **(B) **tumors (200×), **(D) **lungs (400×), and **(F) **bones (400×) (six mice per experimental group and 10 fields per section per organ). Purple-blue staining represents MCs toluidine expression.

However, to confirm further the *in vivo *infiltration of MCs, toluidine staining was also conducted on tissue sections. MCs contain granules (metachromatic) composed of heparin and histamine. Toluidine blue stains MC blue-purple (metachromatic staining) and the background blue (orthochromatic staining). Clearly, in both the arthritic PyV MT and SKG mice, we observe a significantly increased infiltration of MCs within the tumor (Figure 5A, B), lung (Figure 5C, D), and bone (Figure 5E, F). Shown in Figures 2 through 5 are representative MC staining in the tissues from one mouse per experimental group. Also shown in Figure 5B is a magnified image of MC infiltration in the 4T1 tumor section of an arthritic SKG mouse. To quantitate the results from Figures 2 through 5, MCs were counted under the microscope (six mice per experimental group and 10 fields per section per organ), and the mean numbers reflected in the graph.

Most important, we noted that in the non-tumor-bearing arthritic mice (SKG and CII-injected C57BL/6 mice), the number of MCs was increased compared with nonarthritic tumor-bearing Balb/C or PyV MT mice (Figures 3 through 5), indicating that the arthritic milieu by itself can increase the MC population in the bones and lungs, creating a conducive niche that attracts the SCF-expressing tumor cells. The MC expression in the lungs and bones of nonarthritic Balb/C and C57BL/6 mice with no tumor was found to be negligible (data not shown). We therefore believe that once tumor cells populate the bone and lungs, the tumor-derived SCF binds to the c-Kit receptor on MCs and helps in the differentiation, maturation, and survival of MCs, remodeling the microenvironment and further increasing the population of MCs [10] and Figures 2 through 5. Thus, the higher tumor burden and metastases seen in the arthritic mice (Figure 1) may be due to the increase in MC infiltration in the tumors and metastatic niches.

### SCF expression on the tumors

**Figure 6:**
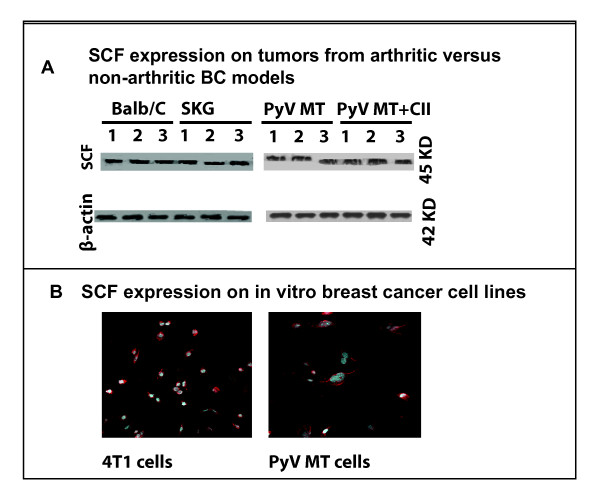
**The expression of stem cell factor (SCF) on the 4T1 and PyV MT cells *in vivo *and *in vitro***. **(A, B) **Western blotting showing the presence of SCF on the tumors derived from experimental mice, as indicated in the figure (three tumors). **(C, D) **Immunofluorescence confirming the presence of SCF on the 4T1 and PyV MT cells *in vitro*.

To confirm that MC activation and proliferation in the tumors and sites of metastasis may be mediated by tumor-derived SCF, we assessed the expression of SCF on 4T1 and PyV MT tumors *in vivo *by Western blotting. We observed that SCF was highly expressed by the 4T1 and PyV MT tumors *in vivo *(Figure 6A). No difference was found in SCF protein expression levels between tumors derived from arthritic versus nonarthritic mice, indicating that the arthritic milieu does not influence SCF levels. SCF expression was also confirmed on 4T1 and PyV MT tumor cell lines *in vitro *by IF (Figure 6B).

### Increased differentiation of MCs from BM-derived hematopoietic precursors in arthritic versus nonarthritic mice with BC

**Figure 7:**
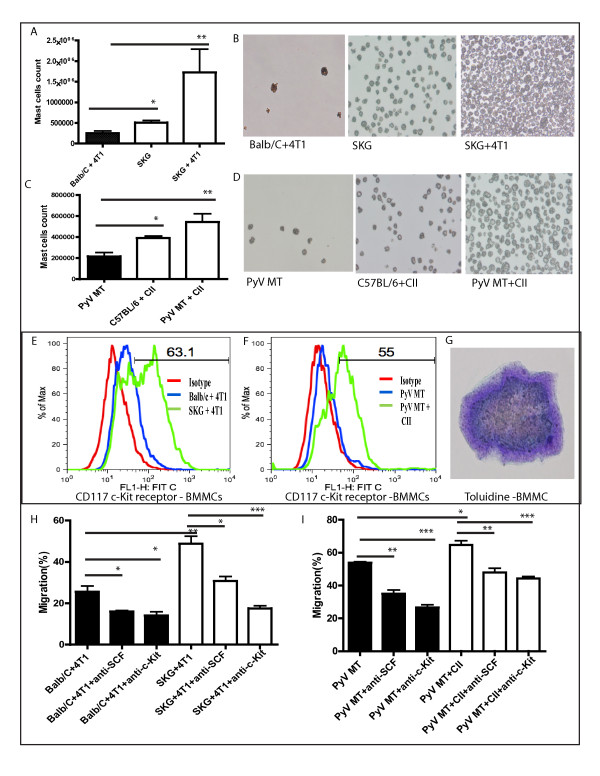
**Increased migration and differentiation**. **(A through G) **Increased differentiation of mast cells (MCs) from bone marrow (BM)-derived hematopoeitic precursors in arthritic versus nonarthritic mice with breast cancer (BC). Significant increase in MC count in **(A) **± tumor-bearing SKG (*P < 0.05; **P < 001) and **(C) **arthritic C57BL/6 and PyV MT (*P < 0.05; **P < 001) mice (10 mice). **(B, D) **Representative images of MCs at 400× magnification. **(E, F) **Flow-cytometric analysis of percentage of cells expressing cKit receptor (CD117) on MCs. **(G) **Representative image of toluidine staining for MCs (1,000× magnification). **(H, I) **Increased migration of tumor cells toward MCs derived from arthritic tumor-bearing mice. Significant increase in migration of 4T1 and PyV MT cells toward the MCs from tumor-bearing arthritic mice (BMMCs) (**P < 001 and *P < 0.05). **(H, I) **Treatment of tumor cells with anti-SCF antibody or MCs with anti-c-Kit antibody decreased the migration of tumor cells toward mast cells. Pretreatment of 4T1 and PyV MT cells with anti-SCF antibody or adding anti-c-Kit antibody to the MCs in the lower chamber significantly decreased the migration of the tumor cells toward the MCs (*P < 0.05; **P < 0.01; ***P < 0.001).

Because we observed increased MCs in the arthritic mice even in the absence of a tumor, we determined whether the differentiation of MCs from the bone marrow-derived precursors was affected by the arthritic milieu. We cultured 2 × 10^6 ^BM cells in the presence of IL-3 and SCF for 30 days to induce the precursor cells to differentiate into MCs. BM cells differentiate into MCs under selection with recombinant IL-3 and SCF [30]. We observed significant increase in the bone marrow-derived mast cells (BMMCs) in non-tumor-bearing arthritic SKG and C57BL/6+CII mice versus Balb/C with tumor or PyV MT mice (Figure 7A and 7C; Tables 1 and 2). Balb/C or C57BL/6 had no detectable levels of mast cells (Table 1 and 2). Numbers of MCs are represented in Figure 7A and 7C, respectively. Light-microscopic images of differentiated MCs from representative groups are shown in Figure 7B and 7D, respectively. Significant increase in MC differentiation was observed in tumor-bearing SKG and arthritic PyV MT mice as compared with tumor-bearing nonarthritic Balb/C mice and nonarthritic PyV MT mice (Figure 7A through D). Because differentiated mast cells should express the c-Kit receptor, we stained the cells with a c-Kit antibody (CD117) and analyzed with flow cytometry. A representative histogram is shown in Figure 7E and 7F. To confirm that the differentiated cells were indeed MCs, toluidine staining was conducted, and representative staining is shown in Figure 7G. Data suggest that the BM-derived precursor cells that are predestined to differentiate into MCs are significantly higher in the arthritic BM milieu before tumors are formed and that the tumors further enhance the differentiation (Tables 1 and 2). It can therefore be speculated that AA probably affects hematopoiesis and induces the production of MCs, thus creating a microenvironment appropriate for tumor cells to home and form metastases.

**Table 1:** Number of bone marrow mast cells generated from bone marrow of Balb/C versus SKG mice induced with breast cancer, stained with toluidine

	Field 1	Field 2	Field 3	Field 4	Field 5	Average	SD
Balb/C 1	0	1	0	1	0	0.4	0.55
Balb/C 1	1	0	0	0	0	0.2	0.45
Balb/C 1	0	0	1	0	1	0.4	0.55
SKG 1	6	4	6	5	7	5.6	1.14
SKG 2	7	5	4	6	8	6	1.58
SKG 3	5	8	7	9	4	6.6	2.07
Balb/C + 4T1 1	0	3	1	0	2	1.2	1.30
Balb/C + 4T1 2	0	0	3	1	0	0.8	1.30
Balb/C + 4T1 3	0	2	0	0	0	0.4	0.89
Balb/C + 4T1 4	1	2	1	0	1	1	0.71
Balb/C + 4T1 5	3	0	0	2	0	1	1.41
Balb/C + 4T1 6	1	0	1	1	0	0.6	0.55
SKG + 4T1 1	18	19	15	14	14	16	2.35
SKG + 4T1 2	17	15	12	17	17	15.6	2.19
SKG + 4T1 3	13	14	15	13	11	13.2	1.48
SKG + 4T1 4	11	13	11	16	15	13.2	2.28
SKG + 4T1 5	18	16	15	18	19	17.2	1.64
SKG + 4T1 6	20	17	21	15	17	18	2.45

**Table 2:** Number of bone marrow mast cells generated from bone marrow of PyV MT mice with and without arthritis, stained with toluidine

	Field 1	Field 2	Field 3	Field 4	Field 5	Average	SD
C57BL/6 1	1	1	0	0	1	0.6	0.55
C57BL/6 2	0	1	0	0	0	0.2	0.45
C57BL/6 3	0	1	1	0	0	0.4	0.55
C57BL/6+CII 1	3	5	4	6	5	4.6	1.14
C57BL/6+CII 2	4	6	5	3	4	4.4	1.14
C57BL/6+CII 3	3	5	5	6	4	4.6	1.14
PyV MT 1	2	3	4	5	5	3.8	1.30
PyV MT 2	4	2	3	4	2	3	1.00
PyV MT 3	3	4	2	3	2	2.8	0.84
PyV MT 4	1	3	2	1	1	1.6	0.89
PyV MT 5	3	2	1	1	3	2	1.00
PyV MT 6	5	1	1	2	0	1.8	1.92
PyV MT+CII 1	13	12	11	12	11	11.8	0.84
PyV MT+CII 2	11	12	11	9	10	10.6	1.14
PyV MT+CII 3	8	14	11	9	7	9.8	2.77
PyV MT+CII 4	10	7	9	13	11	10	2.24
PyV MT+CII 5	9	7	12	11	15	10.8	3.03
PyV MT+CII 6	11	16	13	8	12	12	2.92

### Migration of tumor cells toward mast cells is decreased when tumor cells are treated with anti-SCF or when mast cells are treated with anti-c-Kit antibodies

To determine that the chemotactic migration of tumor cells into metastatic sites is indeed mediated by MCs, and SCF on tumors and c-Kit on mast cells are necessary for this, we conducted an *in vitro *migration assay. SCF expressing 4T1 and PyV MT cells (as shown in Figure 6B) were placed in the upper chamber, and BMMCs that express c-Kit (Figure 7E, F) of arthritic and nonarthritic mice in the lower chamber. We found significant increase in the migration of 4T1 and PyV MT cells toward the MCs that were derived from 4T1-bearing arthritic SKG or PyV MT mice (Figure 7H and 7I, respectively). We further showed that pretreatment of 4T1 and PyV MT cells with anti-SCF antibody or adding anti-cKit antibody to the BMMCs in the lower chamber significantly decreased the migration of the tumor cells toward the MCs (Figure 7H and 7I). The data suggest that the SCF/c-Kit signaling may be one of the drivers of BC-associated bone and lung metastasis.

### Blocking the SCF-cKit interaction significantly decreases the metastasis of 4T1 tumors toward the lungs and bones of SKG mice

**Figure 8:**
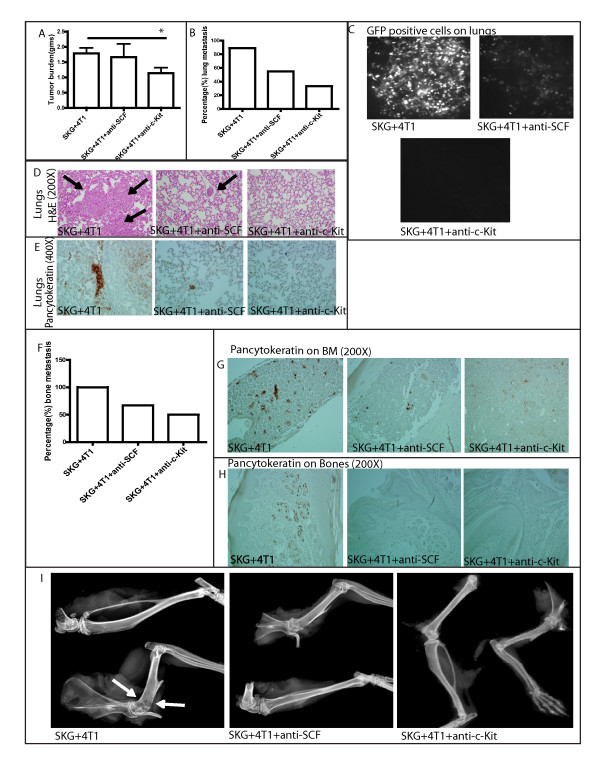
**Diminution of lung and bone metastasis by interrupting the SCF/cKit signaling**. **(A) **Significant decrease in tumor burden in the mice treated with anti-cKit (*P < 0.05). **(B) **Percentage incidence of lung metastasis by blocking the SCF/cKit signaling. **(C) **Representative images of 4T1-GFP cells in lungs of arthritic mice with no treatment. **(D, E) **Representative images of (D) H&E (200×) and (E) pancytokeratin (400×) staining of lungs to confirm micrometastasis. **(F) **Percentage incidence of bone metastasis by blocking the SCF/cKit signaling. **(G, H) **Representative images of pancytokeratin staining of (G) BM (200×) and (H) bone tissue (200×) to confirm metastasis. **(I) **Representative r-ray images of metastatic bone lesions in arthritic model with no treatment. (A through I, 10 mice.) Brown staining represents pancytokeratin positivity.

A key finding from this study indicates that the SCF/c-Kit signaling was necessary for the tumor cells to migrate toward the MCs in an *in vitro *migration assay. This set the stage for examining the effects of blocking this signaling *in vivo*. When mice were treated with therapy to target the c-Kit receptor or SCF, a significant decrease in tumor burden was noted in the mice treated with anti-cKit antibody but not with anti-SCF antibody (Figure 8A). However, metastasis to the lungs (Figure 8B through E) and bone (Figure 8F through I) was significantly reduced in both treatment groups. We observed about threefold and about twofold decrease in lung metastasis in mice treated with anti-cKit and anti-SCF, respectively (Figure 8B). Figure 8C shows representative images of GFP-positive 4T1 cells in the lungs of SKG mice injected with 4T1 cells with no treatment versus the treated groups. Figure 8D shows the metastatic lesions in the lungs and the pancytokeratin staining, confirming that the metastatic patches seen are epithelial tumor cells (Figure 8E). Similarly, we observed about threefold and about twofold decrease in metastatic bone lesions in mice treated with anti-cKit and anti-SCF, respectively (Figure 8F). Pancytokeratin staining in the BM and bone tissues (Figure 8G, H) and representative radiographic x-ray bone images (Figure 8I) confirmed the presence of epithelial tumor cells in the bones of these mice.

## Significant decrease in differentiation of mast cells from bone marrow precursors from the mice treated with anti-cKit and anti-SCF therapy

**Figure 9:**
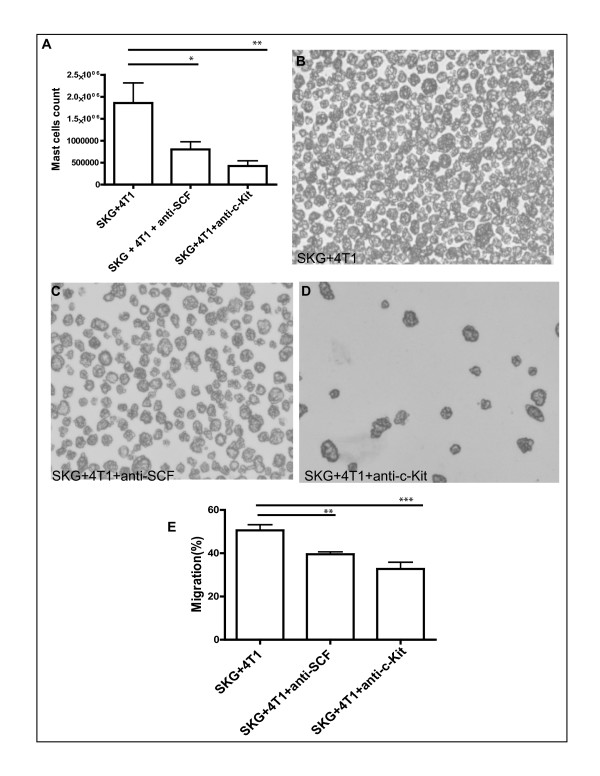
**Decreased differentiation of mast cells (MCs) from bone marrow (BM) precursors in mice treated with anti-SCF or anti- c-Kit antibody**. **(A) **Significant reduction in the differentiation of BMMCs in mice treated with neutralizing stem-cell factor (SCF) or cKit antibodies (*P < 0.05; **P < 0.01). **(B **through **D) **Representative images of BMMCs showing diminution of MC population in treated group (A through D, 10 mice). **(E) **Significant reduction in migration of 4T1 cells toward MCs from mice treated with anti-SCF or anti-cKit (**P < 0.01; ***P < 0.001).

To determine the effect of the therapy on MC differentiation, we plated 2 × 10^6 ^cells from each treatment group of mice. After about 30 days of culture with IL-3 and SCF, we observed a significant decrease in MC population from the BM of mice treated with anti-cKit and anti-SCF (Figure 9A through D), indicating that the BM cells destined to differentiate to MCs are affected by this therapy.

Next, we performed a migration assay by keeping the 4T1 cells in the upper chamber and MCs derived from the mice with and without treatment in the lower chamber. We saw a significant decrease in migration of 4T1 cells toward the MCs derived from the mice treated with anti-cKit and anti-SCF (Figure 9E). This finding illustrates that this therapeutic intervention not only reduces the differentiation of MCs from BM precursors but also affects migration of the tumor cells toward those MCs. In all instances, treatment with anti-c-Kit was significantly better than that with anti-SCF. The reason for this is unknown, but one can speculate that the anti-c-Kit antibody may have a superior neutralizing effect.

### Interrupting the SCF/c-Kit signaling significantly reduces the numbers of mast cells infiltrating the tumor sites

**Figure 10:**
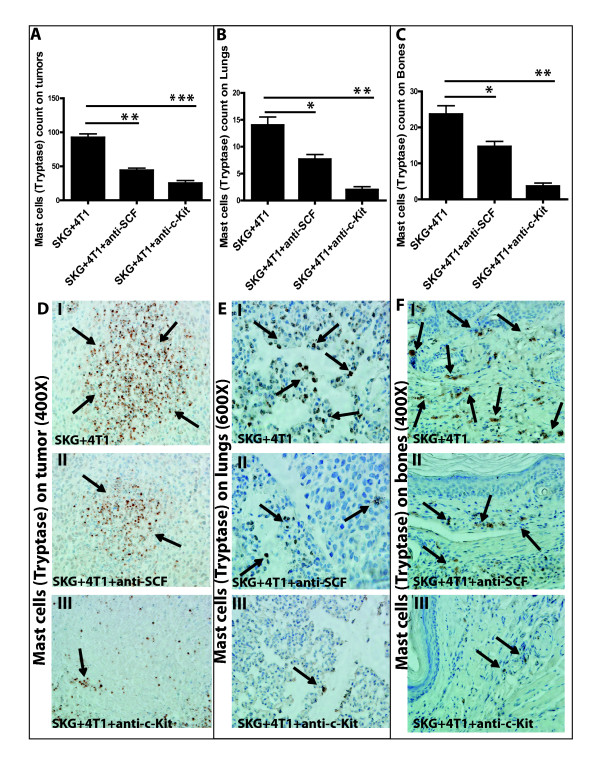
**Significant reduction in number of mast cells (MCs) (tryptase staining) in the tumor, lungs, and bones of mice treated with anti-SCF or anti-ckit antibody**. **(A **through **C) **Significant reduction in number of MCs in (A) tumors (**P < 0.01; ***P < 0.001), (B) lungs (*P < 0.05; **P < 0.01), and (C) bones (*P < 0.05; **P < 0.01) of treated mice. **(D **through **F) **Representative images of MCs tryptase in (D) tumors (400×), (E) lungs (600×), and (F) bones (400× magnification) (six mice per experimental group and 10 fields per section per organ). Brown staining represents MCs tryptase expression.

Finally, to determine the levels of mast cells in the treated mice, we assessed the number of mast cells in the tumor, bone, and lung of treated mice. We observed a significant decrease in MC infiltration in the tumor, lungs, and bones of mice treated with anti-cKit receptor antibody (Figures 10A through C and 11A through C). Representative tryptase and toluidine staining of tumor, lung, and bone tissue sections is shown in Figures 10D through F and 11D through F, respectively. Interestingly, mice treated with anti-SCF also showed significant decrease in MC accumulation in the tumor, lung, and bone (Figure 10D(ii), E(ii), F(ii), and 11D(ii), E(ii), and 11F(ii), respectively), confirming the role of SCF in MC proliferation [10]. Taken together, these findings confirm that the decrease in metastasis in the treated groups (Figure 8) was driven by low MC accumulation.

**Figure 11:**
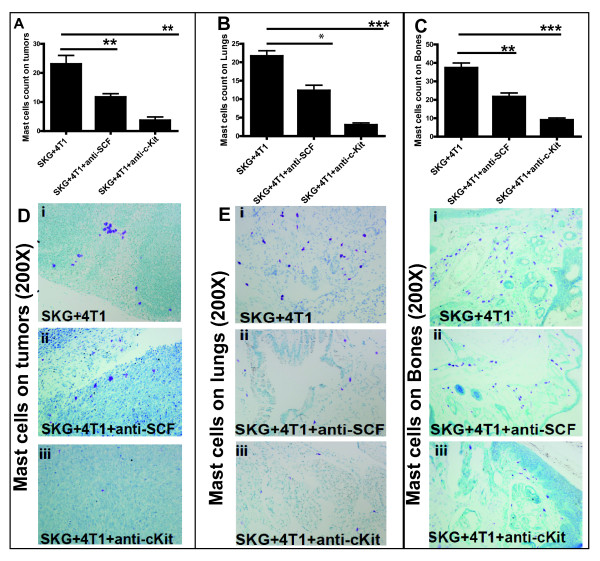
**Significant reduction in number of mast cells (MCs) (toluidine staining) in the tumors, lungs, and bones of mice treated with anti-stem cell factor (SCF) or anti- c-Kit antibody**. **(A **through **C) **Significant reduction in number of MCs in (A) tumors (**P < 0.01), (B) lungs (*P < 0.05; ***P < 0.01), and (C) bones (**P < 0.01; ***P < 0.001) of treated mice. **(D **through **F) **Representative images of MCs toluidine in **(D) **tumors, (E) lungs, and (F) bones (200× magnification) (six mice per experimental group and 10 fields per section per organ). Purple-blue staining represents MCs toluidine expression.

## Discussion

From our previous [8,9] and present study (Figure 1), we confirmed that BC-associated metastasis is significantly augmented in mice with arthritis. Previously, we identified IL-17, IL-6, COX-2, VEGF, MMP-9, and TNF-α as the major underlying factors responsible for the increased metastasis in arthritic mice [8,9]. These cytokines play an important role in cancer development and progression [36-47] and are known to be produced by MCs [10]. Therefore, we analyzed whether MC accumulation and signaling plays a role in augmented BC-associated metastasis.

Data from this study suggests two things:

1. MC population is significantly increased in the bone and lung of arthritic mice before tumor development (Figures 3 through 5). This probably acts as a chemoattractant for breast tumor cells, and once the tumor cells infiltrate the bone and lung microenvironment, they further augment the accumulation of MCs (Figures 3 through 5); and

2. MCs infiltrate the primary tumor site, accumulate there (Figure 2A, B), and possibly increase the metastatic potential of the tumor cells. In the nonarthritic mice, this accumulation of mast cells in the primary tumor as well as in the metastatic niches (bone and lung) is significantly lower than in their arthritic counterparts (Figures 2 through 5), and these mice have significantly fewer metastases (Figure 1). We therefore believe that, in our mouse models, the major driver for the enhanced metastasis is the MCs.

MCs express the receptor, c-Kit, and interact with its ligand SCF expressed on tumor cells. We report that this interaction between MCs and tumor cells is an underlying mechanism for facilitating metastasis in the arthritic mice. Because MCs express the c-Kit receptor, they increase SCF/c-Kit signaling. This sets up a "vicious cycle" whereby SCF/c-Kit signaling further stimulates the production of more MCs, which promotes metastasis. We determined that the BM progenitor cells destined to differentiate into MCs is also significantly increased in the arthritic mice with BC (Figure 7A through G). We established that the BC cells (expressing SCF) migrate toward the MCs expressing c-Kit. This migration was significantly enhanced when MCs were derived from tumor-bearing arthritic mice (Figure 7H, I). Importantly, BC cell migration was significantly decreased when either c-Kit or SCF was blocked by neutralizing antibody (Figure 7H, I).

The *in vitro *data were validated *in vivo *in mice treated with the c-Kit or SCF neutralizing antibodies (Figure 8). We first determined whether metastasis was attenuated by blocking the SCF/cKit interaction. Mice treated with these antibodies had significantly fewer metastases (Figure 8). Second, these mice generated fewer BMMCs (Figure 9A through D), Third, the migration of BC cells toward these MCs (from treated mice) was significantly reduced (Figure 9E). These data confirm that SCF/cKit axis plays a major role in migration of BC cells toward the MCs in the metastatic niches in the arthritic condition.

We recognize that c-Kit is expressed in HSCs, MCs, melanocytes, and germ cells. It is also expressed in hematopoietic progenitor cells including erythroblasts, myeloblasts, and megakaryocytes. However, with the exception of MCs, expression decreases as these hematopoietic cells mature, and c-Kit is not present when these cells are fully differentiated. Simultaneously, MCs have a role in the pathogenesis of RA, and inhibition of c-Kit is reported to inhibit MC activity and also abrogate the contribution of MCs to synovial inflammation in RA [48]. Recently, it was also reported that inhibition of the kit ligand/cKit axis attenuates metastasis in the mouse model, mimicking BC relapse after radiotherapy [49].

It is of note that the effect on the primary tumor was less dramatic, and anti-SCF treatment had no effect on the primary tumor. Along similar lines, anti-SCF treatment was less effective than anti-c-Kit treatment in reducing metastasis. The reason is unknown, and future studies with other combinations will be needed. Similar data were also generated in the PyV MT model (data not shown). These data become relevant, as ckit-SCF interaction is a major agonist for human MC development. It affects proliferation of MC precursors, activation, chemotactic properties, and MC adhesion, and changes MC releasability [50,51]. Therefore, this treatment may have the potential to be developed for treatment of human disease. It is important to note that, even in the nonarthritic BC models (Balb/C with 4T1 tumors and nonarthritic PyV MT tumors), this axis plays a major role (Figure 7H, I).

Our study shows that in our mouse models, MCs seem to be an important regulator, inducing metastasis via the SCF/cKit axis. Therefore we propose that AA creates a proinflammatory microenvironment within the BM and increases the numbers of MC precursors that differentiate into activated MCs expressing the c-Kit receptor. Activated MCs are recruited to the bone and lung, where they accumulate and attract the tumor cells toward those organs.

Concurrently, mast cells also accumulate in the tumor microenvironment. This enables the malignant SCF-expressing BC cells to become more metastatic and circulate in the blood. The circulating BC cells are attracted to leave the circulation when they encounter the ckit receptor on MCs in the bone and lungs. SCF from the tumor cells further triggers the cKit signaling pathway and signals the differentiation, maturation, and survival of MCs, remodeling the microenvironment by intensifying inflammation and releasing the proinflammatory cytokines involved in metastasis [10].

This study may have significant clinical implications. It is reported that women with BC and arthritis have lower survival as compared with women with BC and no arthritis [7]. It is also reported that patients with RA have huge infiltration of MCs [52,53], and MCs promote tumor growth, migration, and invasion [54,55]. We show that interrupting the SCF/cKit-MC signaling significantly reduces BC-associated bone and lung metastases.

## Conclusions

Our data provide clear evidence that MCs play a critical role in remodeling not only the tumor microenvironment but also the metastatic niche to facilitate efficient metastasis through the SCF/cKit interaction, especially in mice with BC and arthritis.

## Abbreviations

AA: autoimmune arthritis; BC: breast cancer; BMMCs: bone marrow-derived mast cells; CIA: collagen-induced arthritis; CII: type II collagen; c-Kit: protooncogene c-Kit or tyrosine-protein kinase Kit or CD117; also called stem cell factor receptor; COX-2: cyclooxygenase-2; IL-6: interleukin-6; IL-17: interleukin-17; MC: mast cell; MMP-9: matrix metallopeptidase-9; MMTV-PyV MT mice: mouse mammary tumor virus-driven polyoma middle-T antigen mice that develop spontaneous mammary gland tumors; RA: rheumatoid arthritis; SCF: stem cell factor; SKG mice: Sakaguchi-developed mice that develop spontaneous arthritis; TNF-α: tumor necrosis factor-alpha; VEGF: vascular endothelial growth factor.

## Competing interests

The authors declare that they have no competing interests.

## Authors' contributions

LDR designed and carried out all the experiments and wrote the manuscript. JMC, MS, DMB, and AK helped with the dissections and end points. HEG interpreted the x-ray imaging. PM is the principal investigator of the laboratory in which the research was performed and contributed to the interpretation of the data and writing of the manuscript. All of the authors read and approved the final manuscript.

## Authors' information

Pinku Mukherjee, PhD, Irwin Belk Distinguished Professor of Cancer Research, Department of Biology, University of North Carolina, Charlotte, NC, USA. Dr Mukherjee has worked on Breast Cancer for the past 22 years.

Lopamudra Das Roy, PhD, Research Assistant Professor, Department of Biology, University of North Carolina, Charlotte, NC, USA. Dr Das Roy has received funding for her work in Breast Cancer Research from the US Department of Defense. Dr Das Roy has immense experience in research with breast cancer and arthritis and received press notice on determining the association between arthritis and breast cancer-associated metastasis.

Jennifer M Curry, PhD, Postdoctoral fellow, Department of Biology, University of North Carolina, Charlotte, NC, USA. Dr Curry has much experience in research with breast cancer. Dr Curry has received funding for her work from the Susan G. Komen Foundation.

Mahnaz Sahraei, PhD, Postdoctoral fellow, Department of Pharmacology, Yale University. Dr Sahraei was graduated from University of North Carolina, Charlotte, NC, USA, under the mentorship of Dr Pinku Mukherjee.

Dahlia M Besmer, graduate student, Department of Biology, University of North Carolina, Charlotte, NC, USA. DMB received funding for her work on breast cancer from the US Department of Defense.

Amritha Kidiyoor, MS, Department of Biology, University of North Carolina, Charlotte, NC, USA.

Helen E. Gruber, PhD, Director, Biology Division, Department of Orthopedic Surgery, Carolinas Medical Center, Charlotte, NC, USA. Dr Gruber has more than 27 years of experience in the area of bone pathology, osteoarthritis, and bone metastasis.

## Supplementary Material

Additional file 1 **The expression of stem cell factor (SCF) on the 4T1 and PyV MT tumors *in vivo *from arthritic BC mice ± anti-SCF treatment**. **(A) **Western blotting showing decreased expression of SCF on the tumors derived from SKG mice with BC and treated with anti-SCF versus no treatment (three to four tumors). **(B) **Graphic representation of the densitometry analysis of SCF expression by using the image J software (**P < 0.01). Click here for file
