# Supplementary figures and images for: Arthritis augments breast cancer metastasis: role of mast cells and SCF/c-Kit signaling
Source: Breast Cancer Res. 2013 Apr 11;15(2):R32. doi: 10.1186/bcr3412 (PMC3672823; doi:10.1186/bcr3412)

**A** SCF expression on tumors from arthritic BC mice  $\pm$  anti-SCF treatment

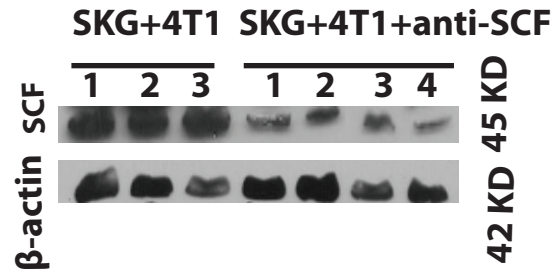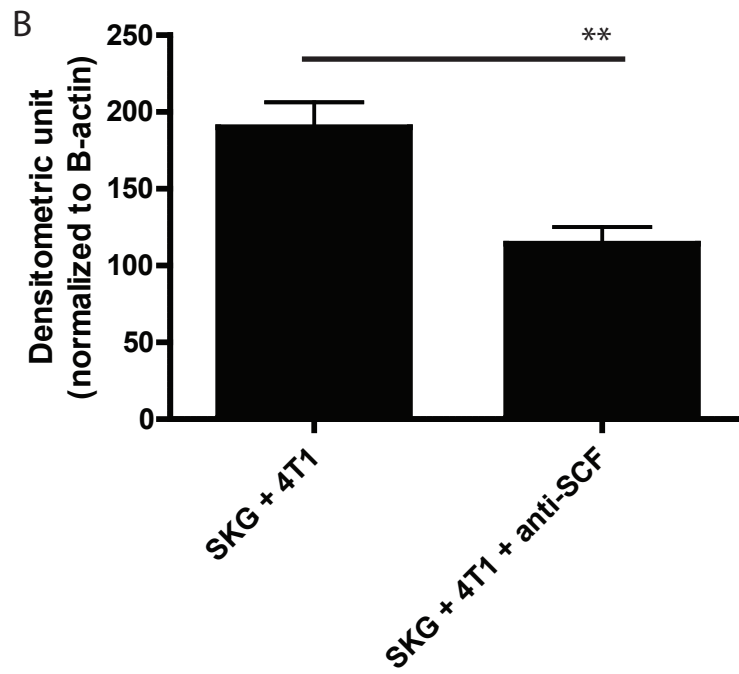

Supplement: Additional file 1 — The expression of stem cell factor (SCF) on the 4T1 and PyV MT tumors in vivo from arthritic BC mice ± anti-SCF treatment. (A) Western blotting showing decreased expression of SCF on the tumors derived from SKG mice with BC and treated with anti-SCF versus no treatment (three to four tumors). (B) Graphic representation of the densitometry analysis of SCF expression by using the image J software (**P < 0.01). [file bcr3412-S1.PDF]
